# Epidemiological characteristics of heatstroke in China, 2010–2023: a longitudinal study based on a national heatstroke surveillance system

**DOI:** 10.1016/j.lanwpc.2025.101722

**Published:** 2025-10-30

**Authors:** Xiaoye Wang, Fan Ding, Xiaoqi Qi, Ziyi Wang, Yingxin Pei, Lijie Zhang, Jinghuan Ren, Yeping Wang, Qing Guo, Biao Zeng, Shiyao Xu, Tian Liu, Rui Wang, Zhifeng Wang, Guoqing Shi

**Affiliations:** a National Key Laboratory of Intelligent Tracking and Forecasting for Infectious Disease, Chinese Center for Disease Control and Prevention, No.155, Changbai Road, Changping District, Beijing, 102206, China; b Chinese Center for Disease Control and Prevention (Chinese Academy of Preventive Medicine), No. 155, Changbai Road, Changping District, Beijing, 102206, China; c Department of Health Policy and Management, School of Public Health, Peking University, No.38 Xueyuan Road, Haidian District, Beijing, 110191, China; d Fujian Provincial Center for Disease Control and Prevention, No.386, Chong'an Road, Jin'an District, Fuzhou City, Fujian Province, 350012, China; e Shenyang Municipal Center for Disease Control and Prevention (Shenyang Health Supervision Institute), No.88, Langming Street, Hunnan District, Shenyang City, Liaoning Province, 110623, China; f Guangdong Provincial Center for Disease Control and Prevention, No.160, Qunxian Road, Panyu District, Guangzhou City, Guangdong Province, 511430, China; g Jingzhou Municipal Center for Disease Control and Prevention, No.6 Qinghe Road, Shashi District, Jingzhou City, Hubei Province, 434000, China

**Keywords:** Heatstroke, Epidemiological monitoring, Public health surveillance

## Abstract

**Background:**

Heatstroke causes numerous pathophysiological changes and can lead to death. Extreme heat events have been increasing in frequency, duration, and intensity worldwide in recent decades and are causing growing public health concern. However, epidemiological studies of heatstroke are limited in number and scope, and have had relatively small sample sizes. To better understand the epidemiological characteristics of heatstroke and advance evidence-based prevention and control strategies, we conducted an observational study of reported heatstroke cases in China.

**Methods:**

Cases included in this study were patients clinically diagnosed with heatstroke during the study period of 2010–2023, reported through a dedicated heatstroke surveillance system established by China CDC. We used descriptive statistical methods to determine the epidemiological characteristics of heatstroke in China.

**Findings:**

There were 86,406 heatstroke cases reported during the study period, with an increasing reporting trend (Spearman correlation coefficient *r* = 0·79). Annual incidence (Spearman *r* = 0·77) and proportion of severe cases (Pearson *r* = 0·67) were positively correlated with the number of nationwide average annual high-temperature days. The overall incidence was 44·83 per 10 million person-years, and the mortality rate was 0·89 per 10 million person-years; 32·02% of cases were severe; the overall case fatality rate (CFR) was 1·98%, and the CFR for severe cases was 6·19%. Most cases and deaths were reported in summer, with peaks in late July. The peaks of incidence, duration, and severity varied by region. The mean age was (50·46 ± 18·73) years, with male cases younger than female cases (49·21 ± 17·41 vs 53·60 ± 21·36; *t* = −28·73, *p* < 0·0001). For every five-year increase in age, the risks of severe heatstroke and death increased by 14·64% (95% *CI*: 1·1415–1·1513) and 25·76% (*CI:* 1·2400–1·2755), respectively. The male:female ratio was 2·49:1; males exhibited a higher risk of suffering from heatstroke and having greater disease severity than females.

**Interpretation:**

Our study provides a clear profile of heatstroke cases, and highlights differences by population, region, and time of year. Results inform formulation of evidence-based strategies for enhanced heatstroke preparedness and response, such as enhancing public health early warning systems, prioritizing protection for vulnerable groups, and advancing localized interventions for risk communication and clinical management.

**Funding:**

Public Health Talent Program of China’s National Disease Control and Prevention Administration.

**Research in context**

**Evidence before this study**

Extreme hot weather is a well-recognized public health concern associated with serious health problems, including heat-related illnesses, acute exacerbations of chronic diseases, heightened health vulnerabilities in special populations, and other indirect hazards. Heatstroke is a direct response of the body when exposed to an extremely hot environment. It is one of the most severe hazards of extreme heat events, which have been increasing as global average and peak temperatures rise. Many studies used mathematical modelling, employing data from outpatient visits or cause-of-death studies, to predict excess deaths during heatwaves. However, the overall health burden of heatstroke is unclear. With an absence of systematic surveillance, there are few nationally-representative, longitudinal epidemiological studies of heatstroke.

**Added value of this study**

This study shows an association of climate change on population health using data from China's purpose-built *Heatstroke Cases Reporting System (HCRS)*. The national surveillance system explicitly links direct health outcomes to climate factors. HCRS offers high-quality data on clinically confirmed cases, in which heatstroke was identified as the primary cause of illness or the immediate cause of death. The large number of cases in our study provides robust attribution of health effects to heat exposure. Our 14-year analysis is the first national-level characterization of heatstroke in China, filling a critical evidence gap on climate-sensitive health outcomes. High-risk temporal patterns, geographic hotspots, and vulnerable demographic groups are identified in the analyses. Findings provide an empirical basis for the development of targeted public health interventions.

**Implications of all the available evidence**

A clear picture of the epidemiological characteristics of heatstroke is essential scientific evidence for public health improvement. Heatstroke is often not considered an illness by the general public, even though risk of heatstroke is increasing in association with global warming. The burden of heatstroke should not be ignored, especially since it can be prevented. There is an urgent need to enhance prevention awareness among the general public and public health professionals to augment response capacity. We found that risk of serious illness and death varies by sex and age, and that the duration of peak heatstroke incidence varies by geographic region. Identifying times, regions, and populations with most risk is an important component of comprehensive heatstroke prevention strategies and action plans to adapt to climate change. Our findings support targeted health education activities for new parents, adult men, and the elderly; establishing cooling facilities and stockpiling medicines based on local environment; and developing and refining health-related early warning models of global warming. Understanding the spatial–temporal distribution of heatstroke is fundamental to effectively target resources and efforts to avert preventable deaths.

## Introduction

Extreme heat is an important environmental and occupational health hazard and a leading cause of weather-related death.^1^ Heatstroke is a medical emergency resulting from exposure to an overly hot environment and is characterized by a cascade of pathophysiological changes. Severe heatstroke has a high case fatality rate (CFR).^1^, ^2^, ^3^ Anthropogenic climate change has driven a global increase in frequency, duration, and intensity of extreme heat events.^4^ China has observed increasing average and peak temperatures, with record highs frequently being surpassed,^5^ particularly during summer and autumn, exacerbating public health burden. In recent years, an increasing number of reports of severe heatstroke have heightened public concern, prompting greater awareness of this pervasive health threat that poses population-wide risks during daily activities. Previous studies provided evidence of heat-related health risks at regional and national scales,^6^, ^7^, ^8^ however the burden of heatstroke remains insufficiently determined, especially in China. While clinical features of heatstroke are well-defined,^9^ epidemiological evidence has been derived predominantly from focused studies with limited observation periods, and highly localized settings, such as individual provinces, cities, or hospitals, and with small sample sizes.^9^, ^10^, ^11^, ^12^, ^13^, ^14^, ^15^, ^16^, ^17^, ^18^, ^19^, ^20^ Longitudinal, multi-regional, large-scale studies of heatstroke are needed to establish population-level risk profiles. Such evidence could serve as a scientific foundation for developing evidence-based action plans with tailored intervention strategies.^21^

In July 2007, China's Ministry of Health and the China Meteorological Administration jointly released the *Health Emergency Response Plan for Heatstroke Incidents (HERP-HI)*, with the intent of understanding the epidemiological characteristics of heatstroke and advancing evidence-based prevention and control strategies and their implementation. Mandated by this policy framework, the Chinese Center for Disease Control and Prevention (China CDC) established a national surveillance system for heatstroke in 2008, which serves as the data platform for this study.^22^ We report results of a longitudinal, epidemiological study of heatstroke in China.

## Methods

### Data sources and data usage

Data on heatstroke cases were extracted from *Heatstroke Cases Reporting System (HCRS)*, a national surveillance system based on the *China Information System for Disease Control and Prevention*, developed by China CDC. Cases in this study were reported by hospitals or local CDCs through HCRS, and reports were reviewed and verified by upper-level CDCs. Cases were individuals who presented to a hospital and were diagnosed with heatstroke between 1 January 2010, and 31 December 2023. Age, sex, disease severity, location of onset, date of onset, date of death, and reporting organization were extracted. We used descriptive analyses to characterize the epidemiological features of reported heatstroke cases.

Population data were obtained from China's 2010 and 2020 National Population Censuses (NPC) and served as denominators for age- and sex-specific incidence and mortality rate calculations.^23^^,^^24^

Nationwide average daily temperature highs were sourced from the China Meteorological Administration's *China Climate Bulletin*.^5^

### Ethical approval

In compliance with ethics exemption requirements of the *Ethical Review Measures for Life Science and Medical Research Involving Human Beings*, jointly issued by the National Health Commission, the Ministry of Education, the Ministry of Science and Technology, and the National Administration of Traditional Chinese Medicine, on February 18, 2023, both ethical approval and need for informed consent were waived.^25^ No patients were contacted; the study was non-interventional; data were anonymized.

### Definitions

Heatstroke cases were reported in accordance with reporting standards specified in China's HERP-HI (2007) guidelines.^22^ Heatstroke is diagnosed based on clinical symptoms, and is directly caused by hot meteorological conditions. Cases with similar symptoms (e.g., fever) caused by other conditions, such respiratory infections or acute gastroenteritis, were excluded.

Heatstroke was classified as mild or severe.^22^ Mild heatstroke was defined as the development of clinical symptoms, such as dizziness, headache, flushing, thirst, profuse sweating, general fatigue, palpitations, rapid pulse, loss of concentration, and uncoordinated movements, along with an increase in body temperature to 38·5 °C (101·3 °F) or higher.^22^

Severe heatstroke was divided into four subtypes: heat cramps, heat exhaustion, heat apoplexy, and mixed presentations. Heat cramps, are pronounced, painful skeletal muscle spasms, predominantly affecting active muscle groups, particularly the gastrocnemius, limb, and abdominal muscles. Contractions are often bilateral and symmetrical, with symptoms intermittently flaring and subsiding. The patient remains fully conscious, and body temperature is usually normal (core temperature 37·5 °C/99·5 °F or lower). Heat exhaustion starts rapidly and is mainly characterized by dizziness, headache, heavy sweating, thirst, nausea and vomiting, followed by cold clammy skin, decreased blood pressure, disturbed heart rhythms, mild dehydration, and with a slightly elevated (below 40 °C/104 °F) or normal body temperature. Heat apoplexy is the most severe and life-threatening of the heat-related conditions. It is characterized by a sudden onset of illness in a hot environment, with a body temperature of 40 °C (104 °F) or higher, profuse sweating in the early stages of the illness, and followed by a period of ‘no sweating’, which may be accompanied by hot, dry skin and varying level of consciousness.^22^

A high-temperature day was defined as a calendar day with a maximum air temperature of 35 °C (95 °F) or higher.^5^^,^^26^, ^27^, ^28^, ^29^

### Data analysis

The analytic database was created with Microsoft Office 2016 software. R version 4·4·2 was used for mapping and statistical analyses. Continental China is divided into seven regions: Northeast, North, Central, South, East, Northwest, and Southwest according to geographical location, topography, climate, hydrology, other geographical, historical, and cultural features.

Continuous variables are reported as mean ± standard deviation *(*SD) or median with interquartile range (IQR). Categorical variables are shown as proportions (%) when describing a distribution. Test levels used α = 0·05 for significance.

Age- and sex-specific incidence and mortality rates were calculated using China national population census data. With the large and relatively slowly changing population in China and the relatively small number of reported cases of heatstroke in hospitals, changes in population size (1·33 billion in 2010–1·41 billion in 2020) had little impact on incidence and mortality rates. Given the authority and credibility of China's NPC, we used data from China's 2010 NPC for denominators in calculations of 2008–2015 rates and data from China's 2020 NPC for denominators in 2016–2023 rates.

Pearson/Spearman correlation analyses were used to evaluate associations between incidence or severity and national annual average number of high-temperature days, and the relationships between age-specific incidence/mortality rates and age (selection based on normality testing). The Kruskal–Wallis rank sum test was used to compare age distributions by severity. Independent sample t-tests were used to compare age differences by sex. Since significant overdispersion was detected in the case data, a negative binomial regression model was used to identify age, sex, and regions associated with heatstroke incidence. Multinomial logistic regression was used to assess how age, sex, and regions were associated with heatstroke severity (mild, severe, fatal), using the mild group as the reference.

### The role of the funder

The funder had no role in study design, data collection, data analysis, data interpretation, writing of the manuscript, or decision to submit.

## Results

Heatstroke has been systematically reported to HCRS since 2008 in China. As of 31 December 2023, there were 87,293 heatstroke cases recorded in HCRS. During 2008–2009, case reporting was incomplete due to the early-stage deployment of HCRS, as there were training sessions for healthcare and CDC professionals and refinement of the digital reporting infrastructure at that time. Data from these two years were excluded from the analytic data set, making the study period 2010–2023.

### Overall situation

During the 2010–2023 study period, 86,406 heatstroke cases were reported; 58,735 (67·98%) were mild and 27,671 (32·02%) were severe. Among severe cases, 47·23% (13,070/27,671) were heat apoplexy, 34·49%, (9545/27,671) were heat cramps, 11·26%, (3117/27,671) were heat exhaustion, and 5·54% (1533/27,671) were mixed. The overall CFR was 1·98%, and the CFR for severe cases was 6·19%. On average, 6172 heatstroke cases and 122 heatstroke deaths were reported each year. The overall incidence was 44·83 per 10 million person-years, with a mortality rate of 0·89 per 10 million person-years. Among severe cases, incidences in descending order were: heat apoplexy (6·78 per 10 million person-years), heat cramps (4·95 per 10 million person-years), heat exhaustion (1·62 per 10 million person-years), and mixed (0·80 per 10 million person-years). Heat apoplexy had the highest mortality rates (0·58 per 10 million person-years), followed by heat exhaustion (0·15 per 10 million person-years), mixed (0·10 per 10 million person-years), and heat cramps (0·02 per 10 million person-years).

**Fig. 1:**
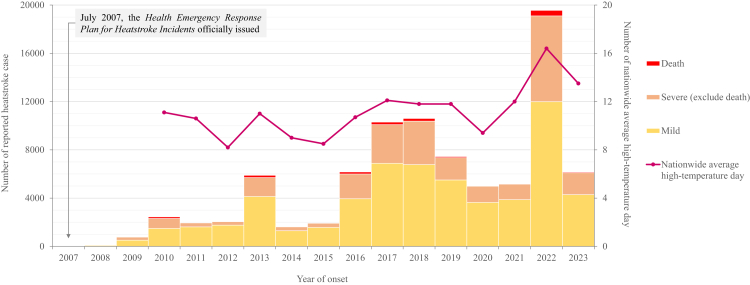
**Annual distribution of reported heatstroke cases by severity and nationwide high-temperature days, 2008–2023, China**.

There was an increasing trend in the number of reported cases (Spearman *r* = 0·7943, *p* = 0·0007). The annual incidence (Spearman *r* = 0·7723, *p* = 0·0012) and the proportion of severe cases (Pearson *r* = 0·6695, *p* = 0·0088) were positively correlated with the number of nationwide average annual high-temperature days (Fig. 1).

### Temporal distribution

**Fig. 2:**
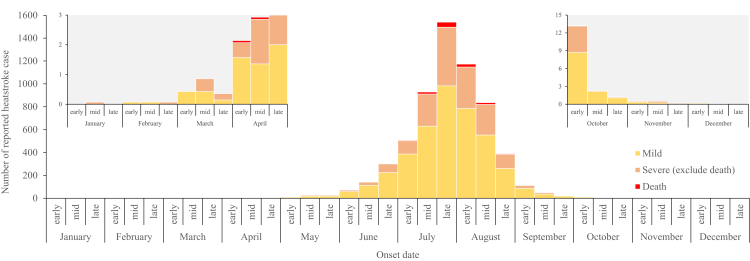
**Average monthly distribution of reported heatstroke cases, 2010–2023, China**.

In China, heatstroke was reported throughout the year, with the highest reporting during June to September. There was a rapid increase starting late June, peaking in late July, and declining thereafter; 87·11% (75,271/86,406) of cases and 94·17% (1614/1714) of deaths were reported in July or August, which also had the highest CFR, averaging 2·15% (Fig. 2).

### Regional distribution

Heatstroke was reported from all seven geographic regions in China. East China reported 78·02% (4815/6172) of the cases, and had the highest incidence (117·34 per 10 million person-years). Central China incidence was 27·27 per 10 million person-years, and Southwest China incidence was 21·93 per 10 million person-years; 94·87% (5856/6172) of cases were reported from these three regions.

**Fig. 3:**
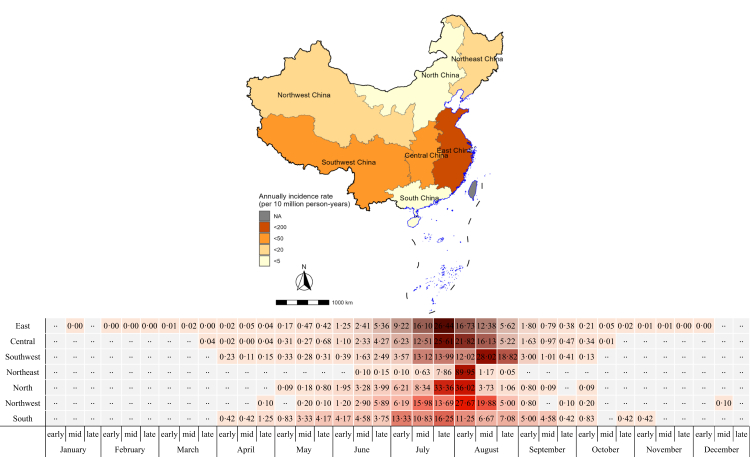
**Heat map of regional and temporal distribution of reported heatstroke cases in the seven geographical regions of China, 2010–2023**. ∗ Numerical values represent the proportion (%) of the total number of reported cases for each region throughout the year.

The occurrence and the duration of high-incidence periods varied by region. South China high incidence started in early July and stayed elevated until early August. East and Central China peaks occurred in late July. North China peak reporting was from late July to early August. Northeast and Northwest China had peaks in early August, while the Southwest China peak was in mid-August (Fig. 3).

### Population distribution

Annual incidence (Spearman *r* = 0·9772, *p* < 0·0001) and mortality (Spearman *r* = 0·9930, *p* < 0·0001) increased with age, with the lowest rates observed in children under 10 years of age (1·91 and 0·02 per 10 million person-years, respectively). The highest rates were among individuals 90 years or older (246·52 and 15·01 per 10 million person-years, respectively).

The average age of reported heatstroke cases was (50·46 ± 18·73) years, ranging from infancy to 103 years. Severity varied by age, with highest severity in older ages (Kruskal–Wallis H = 4340·6, *p* < 0·0001). Post-hoc analysis revealed that mild cases (n = 58,735) were younger on average (48 years, IQR 33–59), than severe cases (n = 25,957; 55 years, IQR 44–70; *Z* = −60·2501, *p* < 0·0001). Fatal heatstroke cases (n = 1714) were the oldest (63 years, IQR 50–77), statistically significantly different from severe cases (*Z* = 13·5890, *p* < 0·0001).

The male-to-female ratio of heatstroke was 2·49:1. Male cases were younger on average than female cases (49·21 ± 17·41 vs 53·60 ± 21·36 years; *t* = −28·7258, *p* < 0·0001). Peak incidence was 50–54 years for both males and females. Among individuals aged 55 years or older, male cases declined rapidly in overall reported cases, severe cases, and fatal cases. Female cases plateaued in overall reported cases with minor fluctuations, while severe and fatal cases increased until age 85, and declined thereafter.

**Fig. 4:**
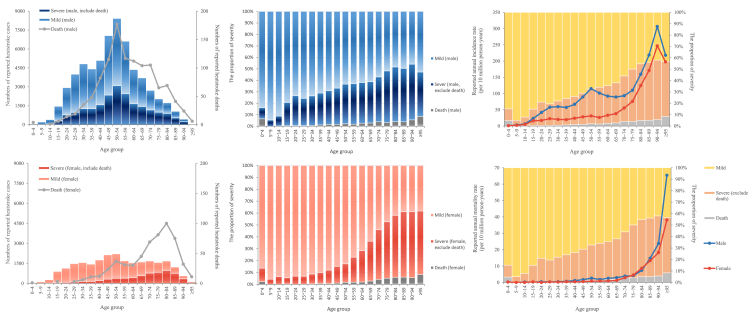
**Age and sex distribution by severity of heatstroke cases reported in China, 2010–2023**.

There were similar trends in males and females for annual incidence and mortality rates. Males had a higher disease burden than females across all age groups, with the male-female disparity peaking in 20–70-year-olds, especially in 50–54-year-olds. Among 20–70-year-old men, cases, incidence, mortality, and proportions of severe cases were 3·10, 2·99, 4·00, and 1·95 times corresponding values in women (Fig. 4).

### Age, sex, and geographic region associations with incidence and severity

Negative binomial regression identified factors associated with heatstroke. Among the variables included in the analysis, sex, age, and geographic region were the most statistically significantly associated with incidence and severity (*p* < 0·0001). A significant age-dependent increase in incidence rate ratios (IRR) was evident, with individuals over 90 years old having incidences more than 300 times greater than a reference group of 0-4-year-olds. Males had 2·28 times higher risk than females (IRR = 2·2770, 95% *CI*: 1·9982–2·5949). There was significant geographic variation: with the Northeast as a reference, East China had the highest risk (IRR = 6·6412, 95% *CI*: 5·2359–8·4231) and South China had the lowest (IRR = 0·0534, 95% *CI*: 0·0401–0·0710) (Fig. 3).

Multinomial logistic regression was used to evaluate independent associations between demographic factors and severity of heatstroke. The regression model included age (as a continuous variable), sex (reference category: female), and geographic region (reference category: East China) as covariates. Odds ratios (ORs) with 95% confidence intervals (CIs) were derived by exponentiating regression coefficients. Age was identified as a significant factor influencing whether heatstroke progressed in clinical severity to severe or fatal. With every five-year increase in age, the risk of severe heatstroke increased by 14·64% (OR = 1·1464, 95% *CI:* 1·1415–1·1513), and the risk of death increased by 25·76% (OR = 1·2576, 95% *CI:* 1·2400–1·2755). Male patients had a higher risk of progressing to severe disease compared to females (OR = 1·7298, 95% *CI*: 1·6698–1·7919). The risk of death was also higher among males than females (OR = 1·2298, 95% *CI*: 1·1066–1·3667), although with a lower OR than for severity. Geographically, using East China as the reference group, North China had the highest risks for both severe heatstroke (OR = 2·5630, 95% *CI:* 2·2623–2·9037) and death (OR = 6·7666, 95% *CI*: 5·3223–8·6029). For severe heatstroke, regional risks in descending order after North China were Northwest (OR = 2·1051, 95% *CI*: 1·8448–2·4021), South (OR = 1·7250, 95% *CI*: 1·3406–2·2196), Southwest (OR = 1·5939, 95% *CI*: 1·5070–1·6859), Northeast (OR = 1·3877, 95% *CI*: 1·2636–1·5238), and Central China (OR = 1·3831, 95% *CI*: 1·3169–1·4526). For heatstroke deaths, regional risks in descending order after North China were Northwest (OR = 2·6278, 95% *CI*: 1·8238–3·7863), Southwest (OR = 2·0489, 95% *CI*: 1·7583–2·3874), and Central China (OR = 1·5575, 95% *CI*: 1·3284–1·8262). No statistically significant difference in mortality risk were found in either Northeast or South China when compared to East China (*p* > 0·05).

## Discussion

During 1990–2019, there has been a steady global increase in the number of heatwave events and excess heat-related deaths, with almost half of these events and deaths occurring in Asia. China has had the second largest burden of excess heatwave deaths, after India.^30^ Climate change is creating unprecedented health threats by amplifying extreme heat exposure in daily-routine and occupational environments. Among the many health hazards caused by heatwaves, heatstroke is the most closely associated with hot weather–and is one of the most preventable of the hazards through use of simple daily precautions. However, if heatstroke is not recognized early in its course, it has a high CFR.^31^ Understanding the epidemiological patterns of heatstroke, including high-risk periods, geographic hotspots, and vulnerable populations can enable public health systems to develop evidence-based interventions, optimize preparedness for prevention, improve control and clinical management, and ultimately reduce the incidence of heatstroke and its preventable mortality.^1^^,^^21^

Heatstroke incidence and severity closely track hot weather conditions. In China, heatstroke is reported throughout the year, with peak reporting in late July and early August. During May and June, the number of cases, severe cases, deaths, proportion of severe cases, and CFR are higher than during September and October. A possible explanation for this phenomenon is inadequate physiological acclimatization and risk awareness to abrupt ambient temperature increases that typically occur in early summer.^11^

Our evaluation found regional disparities in heatstroke epidemiology. The peak timing and incidence of heatstroke had south-to-north and east-to-west gradients. The temporal characteristic of heatstroke peaks in China had distinct regional patterns, with earliest occurrence and prolonged persistence observed in the East and South relative to the North and West regions. East China had the highest incidence but the lowest severity. This may be attributable to the region's high population density, advanced economic development, strong health literacy among residents, and superior medical services, leading to increased medical attention seeking behavior and increased reporting of medically-attended events, therefore increasing incidence but decreased severity assessments. South China had the lowest incidence, but had prolonged periods of relatively high incidence, resulting in a marked plateau in heatstroke case reports. This pattern generally aligns with the occurrence of sustained regional high temperatures in South China from mid-May to early-October.^5^^,^^32^ Other reasons include greater adaptability of individuals used to living in hot weather for a long time, and more time-honored heatstroke prevention methods developed and used by local residents–for example, consuming herbal cooling beverages brewed from Chinese medicinal herbs, and dietary incorporation of specific botanicals for heat-related illness mitigation. Regional disparities in severe and fatal outcomes could be attributed to variations in climatic conditions, healthcare infrastructure, population acclimatization, and public awareness of preventive measures. These potential influencing factors could be topics for further research.

We observed significant disparities in incidence and clinical severity by age and sex. Both incidence and severity rates markedly increased with advancing age, particularly among people over 70 years old. This elevated risk likely relates to age-related physiological vulnerabilities and behavioral tendencies among the elderly, including higher prevalence of underlying medical conditions, less use of air conditioning, reduced capacity for activities of daily living, and increased dependency on caregivers. These vulnerabilities may lead to delayed recognition, less healthcare use, and fewer clinical interventions–all well-recognized risk factors for heatstroke.^9^^,^^31^ Males aged 20–70 years represent another critical high-risk population necessitating targeted heatstroke mitigation strategies, particularly among 45–60-year-olds–the most skilled labor force. Elevated vulnerability may contribute to occupational exposures and suboptimal protective behaviors.^19^ A finding of interest is that the CFR in women is higher than in men. This may be due to women's longer life span, as seen in the greater proportion of cases among older women than older men. Since CFR is higher in older individuals regardless of sex, the finding is likely related to longer life spans in females.

Our study has limitations. First, cases were patients who presented to hospitals and were clinically diagnosed with mild or severe heatstroke. Heatstroke with aura, or community cases not seeking medical care were unable to be included. More than 70% of people who experience heat-related symptoms do not seek medical care.^34^ This would result in fewer less severe cases being included, which would underestimate the full burden of heatstroke in China and overestimate CFR. However, severe or worse cases would most likely seek medical attention. Second, due to constraints in the initial design of the case reporting form, occupational and exposure histories, timing of detection, and measures of intervention and treatment were unavailable. Consequently, factors contributing to severe heatstroke could not be addressed. The surveillance system was upgraded in 2023 to include occupational, exposure, and some clinical data, which will be used in future studies with this system. The surveillance system does not record ethnicity, precluding analysis by ethnicity.

Heatstroke can affect anyone and can lead to death. However, much of the mortality and heat-related health risks are preventable. Evidence-based action plans that integrate behavioral interventions and biophysical mitigation strategies are needed.^21^ The public should be fully aware of potential health hazards and severity of heatstroke.^33^ Rapid cooling is the primary treatment for severe heatstroke. Early detection, early removal from the hot environment, early diagnosis, and early treatment can effectively improve heatstroke resuscitation success rates and clinical outcomes for severe heatstroke.^2^^,^^20^ Public health authorities should use evidence-based risk communication campaigns to bolster community capacity for heatstroke prevention, recognition, self-response, and bystander intervention, with targeted outreach to high-risk populations informed by vulnerability mapping analyses.^34^ There are many factors influencing vulnerability, including geographical and demographic heterogeneity in thermal sensitivity threshold and heat adaptive capacity; health effects had lagged associations with thermal exposures.^9^^,^^35^, ^36^, ^37^, ^38^ We recommend developing a more sensitive health-related early warning system for heatstroke by implementing context-specific risk modeling frameworks.^39^ A dynamic, precise heatstroke health index, that delivers timely alerts with localized and personalized risk stratification through validated predictive models can empower communities to interpret exposure threats immediately and take effective preparedness and response actions.

In 2008, the Chinese government launched a call for nationwide reporting of heatstroke cases through a standardized national surveillance system with unified case definitions and a digital reporting portal. This initiative systematically captured demographic characteristics and clinical severity profiles of heatstroke patients and provided a practical approach for monitoring health hazards in the context of climate change. Using these longitudinal, multi-regional, and large-scale data, our study provides a picture of the epidemiological characteristics of heatstroke cases and captures fundamental trends of heatstroke in China. Enhanced effort is still needed and recommended, including, but not limited to continue advancing surveillance quality through improving clinician awareness in detection and reporting, establishing self-reporting channels, undertaking community surveys and estimating the magnitude of non-care-seeking heatstroke cases, conducting case investigations regarding to pre-onset exposure profiles, illness progression and medical management processes, and performing environmental epidemiological research. These measures will further clarify the disease burden and identify risk factors for severe cases and deaths.

The health impacts of extreme heat are multifaceted, ranging from immediate short-term health consequences to persistent long-term health effects that threaten overall wellbeing. Rising global temperatures drive more intense heatwaves, with heatstroke emerging as one of the leading weather-associated health hazards. As the human body's most easily recognized thermoregulatory response, heatstroke could be considered to serve as a critical biological indicator for broader heat-related health hazards. Heatstroke surveillance could enable predictive modeling of associated health risks and inform public health adaptation strategies. Research of epidemiological patterns of heatstroke can facilitate improvement of evidence-based interventions for control and prevention, offering valuable insights and replicable models for mitigating acute and chronic health impacts across diverse extreme weather scenarios while advancing multi-hazard prevention capabilities. Adopting a “One Health” perspective, which operationalizes the interconnectedness of human, animal, and environmental health, extreme heat acts as both a direct cause of human health hazards and an exacerbating factor in anthropogenic environmental degradation, creating a reinforcing cycle that increases ecological and public health risks.^38^, ^39^, ^40^, ^41^, ^42^, ^43^, ^44^ Beyond defensive adaptation, collective climate mitigation is now the primary preventive medicine for long-term health security and environmental viability.

## Contributors

Conceptualisation: Guoqing Shi.

Methodology: Xiaoye Wang, Fan Ding, Yingxin Pei, and Lijie Zhang.

Data Curation: Xiaoye Wang and Fan Ding.

Formal Analysis: Xiaoye Wang, Fan Ding, Biao Zeng, Xiaoqi Qi, and Ziyi Wang.

Validation: Xiaoye Wang, Fan Ding, Xiaoqi Qi, and Ziyi Wang.

Visualisation: Xiaoye Wang, Ziyi Wang, and Tian Liu.

Original Draft: Xiaoye Wang, Xiaoqi Qi, and Ziyi Wang.

Review and editing: Xiaoye Wang, Fan Ding, Yingxin Pei, Lijie Zhang, Biao Zeng, Shiyao Xu, Yeping Wang, Zhifeng Wang, and Guoqing Shi.

Resources: Guoqing Shi.

Project Administration: Xiaoye Wang, Fan Ding, Jinghuan Ren, Guo Qing, and Rui Wang.

Supervision: Guoqing Shi.

Decision-making for submission: Guoqing Shi.

Funding Acquisition: Guoqing Shi.

The authors had unrestricted access to the study data, approved the final manuscript, and retained ultimate responsibility for the decision to submit the paper for publication.

## Data sharing statement

Case definitions and reporting protocols in this study are available at https://www.gov.cn/zwgk/2007-07/31/content_701832.htm. The meteorological information was obtained from https://www.cma.gov.cn/zfxxgk/gknr/qxbg/. All individual data used in this study are available upon reasonable request. For more information on data access, contact Xiaoye Wang (wangxy2@chinacdc.cn).

## Editor note

The Lancet Group takes a neutral position with respect to territorial claims in published maps and institutional affiliations.

## Declaration of interests

Authors declare no competing interests.
