# Corrigendum to “Epidemiological characteristics of heatstroke in China, 2010–2023: a longitudinal study based on a national heatstroke surveillance system” [The Lancet Regional Health – Western Pacific 64C (2025) 101722]

**DOI:** 10.1016/j.lanwpc.2025.101778

**Published:** 2025-12-09

**Authors:** Xiaoye Wang, Fan Ding, Xiaoqi Qi, Ziyi Wang, Yingxin Pei, Lijie Zhang, Jinghuan Ren, Yeping Wang, Qing Guo, Biao Zeng, Shiyao Xu, Tian Liu, Rui Wang, Zhifeng Wang, Guoqing Shi

**Affiliations:** a National Key Laboratory of Intelligent Tracking and Forecasting for Infectious Disease, Chinese Center for Disease Control and Prevention, No.155, Changbai Road, Changping District, Beijing, 102206, China; b Chinese Center for Disease Control and Prevention (Chinese Academy of Preventive Medicine), No. 155, Changbai Road, Changping District, Beijing, 102206, China; c Department of Health Policy and Management, School of Public Health, Peking University, No.38 Xueyuan Road, Haidian District, Beijing, 110191, China; d Fujian Provincial Center for Disease Control and Prevention, No.386, Chong'an Road, Jin'an District, Fuzhou City, Fujian Province, 350012, China; e Shenyang Municipal Center for Disease Control and Prevention (Shenyang Health Supervision Institute), No.88, Langming Street, Hunnan District, Shenyang City, Liaoning Province, 110623, China; f Guangdong Provincial Center for Disease Control and Prevention, No.160, Qunxian Road, Panyu District, Guangzhou City, Guangdong Province, 511430, China; g Jingzhou Municipal Center for Disease Control and Prevention, No.6 Qinghe Road, Shashi District, Jingzhou City, Hubei Province, 434000, China

The authors regret <the Funding section should be stated as: Public Health Talent Program of China’s National Disease Control and Prevention Administration (Grant No. 01064); Operation of Public Health Emergency Response Mechanism (Grant No. 102393220020010000017).>.

The authors would like to apologise for any inconvenience caused.

